# Gamma Radiation-Mediated Synthesis of Antimicrobial Polyurethane Foam/Silver Nanoparticles

**DOI:** 10.3390/polym16101369

**Published:** 2024-05-10

**Authors:** Eduard-Marius Lungulescu, Radu Claudiu Fierascu, Miruna S. Stan, Irina Fierascu, Elena Andreea Radoi, Cristina Antonela Banciu, Raluca Augusta Gabor, Toma Fistos, Luminita Marutescu, Marcela Popa, Ionela C. Voinea, Sorina N. Voicu, Nicoleta-Oana Nicula

**Affiliations:** 1 National Institute for Research and Development in Electrical Engineering ICPE-CA, 313 Splaiul Unirii, 030138 Bucharest, Romania; marius.lungulescu@icpe-ca.ro (E.-M.L.); elena.radoi@icpe-ca.ro (E.A.R.); cristina.banciu@icpe-ca.ro (C.A.B.); 2 National Institute for Research & Development in Chemistry and Petrochemistry—ICECHIM, 202 Spl. Independentei, 060021 Bucharest, Romania; fierascu.radu@icechim.ro (R.C.F.); irina.fierascu@icechim.ro (I.F.); raluca.gabor@icechim.ro (R.A.G.); toma.fistos@icechim.ro (T.F.); 3 Faculty of Chemical Engineering and Biotechnology, National University of Science and Technology Politehnica Bucharest, 1-7 Gh. Polizu Str., 011061 Bucharest, Romania; 4 Department of Biochemistry and Molecular Biology, Faculty of Biology, University of Bucharest, 91-95 Splaiul Independentei, 050095 Bucharest, Romania; miruna.stan@bio.unibuc.ro (M.S.S.); sorina.voicu@bio.unibuc.ro (S.N.V.); 5 Faculty of Horticulture, University of Agronomic Sciences and Veterinary Medicine of Bucharest, 59 Marasti Blvd., 011464 Bucharest, Romania; 6 Department of Microbiology, Faculty of Biology, University of Bucharest, 91-95 Splaiul Independentei, 050095 Bucharest, Romania; luminita.marutescu@bio.unibuc.ro (L.M.); marcela.popa@bio.unibuc.ro (M.P.)

**Keywords:** polyurethane nanocomposites, silver nanoparticles, antimicrobial, gamma rays

## Abstract

Nosocomial infections represent a major threat within healthcare systems worldwide, underscoring the critical need for materials with antimicrobial properties. This study presents the development of polyurethane foam embedded with silver nanoparticles (PUF/AgNPs) using a rapid, eco-friendly, in situ radiochemical synthesis method. The nanocomposites were characterized by UV–vis and FTIR spectroscopy, scanning electron microscopy coupled with energy dispersive X-ray technique (SEM/EDX), differential scanning calorimetry (DSC), dynamic mechanical analysis (DMA), tensile and compression strengths, antimicrobial activity, and foam toxicity tests. The resulting PUF/AgNPs demonstrated prolonged stability (over 12 months) and good dispersion of AgNPs. Also, the samples presented higher levels of hardness compared to samples without AgNPs (deformation of 1682 µm for V1 vs. 4307 µm for V0, under a 5 N force), tensile and compression strength of 1.80 MPa and 0.34 Mpa, respectively. Importantly, they exhibited potent antimicrobial activity against a broad range of bacteria (including *Pseudomonas aeruginosa*, *Staphylococcus aureus, Escherichia coli*, and *Enterococcus faecalis*) and a fungal mixture (no fungal growth on the sample surface was observed after 28 days of exposure). Furthermore, these materials were non-toxic to human keratinocytes, which kept their specific morphology after 24 h of incubation, highlighting their potential for safe use in biomedical applications. We envision promising applications for PUF/AgNPs in hospital bed mattresses and antimicrobial mats, offering a practical strategy to reduce nosocomial infections and enhance patient safety within healthcare facilities.

## 1. Introduction

One of the most significant challenges facing the healthcare system is the widespread occurrence of nosocomial infections, also known as a healthcare-associated infection (HAI) [1]. These infections, caused by various pathogens such as bacteria, viruses, and fungi, are frequently associated with factors like invasive medical procedures, prolonged hospital stays, and compromised immune systems. Moreover, nosocomial infections pose significant risks, potentially leading to severe complications like sepsis and mortality. Often, these infections are induced by multidrug-resistant pathogens acquired through invasive procedures, inappropriate antibiotic use, and inadequate adherence to infection control protocols [1,2,3].

Antimicrobial materials are very important in preventing the spread of infections and diseases by aiding in infection control, preventing hospital-acquired infections, and facilitating surface disinfection [4,5]. Furthermore, they offer a promising solution to combat antibiotic resistance with their ability to inhibit microbial growth [6,7].

Polyurethane foam finds many biomedical applications due to its favorable mechanical properties and biocompatibility, including the manufacturing of stents, vascular prostheses, breast implants coatings, dressings, antibacterial surfaces, catheters, etc. [8,9]. Nonetheless, the cytotoxicity associated with the raw materials may restrict its utilization in specific applications, requiring meticulous attention to the isocyanate index in order to ensure biocompatibility [10]. In a particular case, the hospital bed mattresses made also from polyurethane foam can be contaminated by microorganisms colonizing the patient’s skin, body fluids such as urine and wound exudates, or feces. Mattresses with a high microbial load can contribute to the horizontal transmission of microorganisms between patients and other surfaces [11,12].

Inducing antimicrobial properties into polyurethane can be achieved through various methods, including the integration of different chemicals [8,13,14] (e.g., quaternary ammonium and polyhexamethylene guanidine compounds as well as metal nanoparticles) or natural products [15,16,17] (e.g., terpenes, plant extracts, and biopolymers) directly into the polyurethane structure. The introduction of metal nanoparticles into the polyurethane foam structure represents an approach for the induction of antimicrobial properties, improving structural and mechanical strength properties, to address various challenges in sectors such as healthcare, food packaging, and materials engineering [8,18]. Moreover, metal nanoparticles are efficient against antibiotic resistant strains and compromise the development of new resistant strains by causing cellular oxidative damage through the generation of reactive oxygen species [19].

Various metallic nanoparticles, including silver (Ag), copper oxide (CuO), zinc oxide (ZnO), titanium dioxide (TiO_2_), gold (Au), etc., have been extensively researched for their antimicrobial properties in polyurethane foams [8,20]. These nanoparticles can be synthesized through diverse methods (chemical, physical, and biological) [8,21,22,23], either externally (ex situ), followed by their introduction into one of the primary components of polyurethane foams (e.g., polyol), or formed within the foam structure by reducing precursor ions (in situ) [8,24]. Each synthesis method has its advantages and drawbacks: (i) Chemical methods enable precise control over particle properties but may involve toxic chemicals and generate potential hazardous wastes; (ii) physical methods offer simplicity and scalability but may require specialized equipment and high energy input, and (iii) biological methods are environmentally friendly and economically viable but may lack control over particle size and shape [25].

This paper presents, for the first time in the literature, to our knowledge, the radiochemical synthesis of nanocomposites composed of polyurethane foam and silver nanoparticles. This synthesis is achieved through gamma ionizing radiation directly within the polyol, uniquely combining both in situ and ex situ principles. The resulting nanocomposites were thoroughly characterized in terms of their optical, morpho-structural, thermal, and mechanical properties. They demonstrated significant antibacterial and antifungal activity, coupled with low toxicity. These results underscore the potential of these materials for a variety of biomedical applications. Notably, they could be used in the production of hospital mattresses, antimicrobial coatings, and in the development of innovative antimicrobial polyurethane foam-based materials such as paints and textiles.

## 2. Materials and Methods

### 2.1. Chemicals

*Metal salt precursor*: Silver nitrate (Mw = 189.87 g/mol, ≥98%, Sigma Aldrich, Saint Louis, MO, USA); *polyether polyol*: Voranol CP3001 (Mw: 3000, OH value: 54–59, Dow Chemical, Wilmington, DE, USA); *catalysts*: 1,4-Diazabicyclo [2.2.2]octane (DABCO 33-LV, Sigma Aldrich, USA) and Dibutyltin dilaurate (Sigma Aldrich, USA); *surfactant*: Vorasurf DC 2585 (Dow chemical, DE); *chain extender*: 1,4-Butanediol (BDO, 99%, Sigma Aldrich, USA); isocyanate: 4,4′-Methylene diphenyl diisocyanate prepolymer (ISO 137/28) (NCO: 18%, BASF, Ludwigshafen, DE, USA); NPs stabilizers: Polyvinylpyrrolidone (PVP, Mw = 360.000, Sigma Aldrich, USA) and sulphate sodium salt (SDS, Mw = 288.38 g/mol, Ph Eur, Sigma Aldrich, USA). These substances were used as received, without further purifications.

Ultrapure water (18.6 MΩ produced by Simplicity UV Water Purification System, Milli-Q, Merck, Burlington, VT, USA) was used both as solvent and blowing agent of polyurethane foam.

### 2.2. Preparation of Polyurethane Foam (PUF)/Ag NPs

To obtain the polyol/AgNPs solution, a quantity of silver nitrate, both with and without stabilizing agents, was dissolved in a volume of water (i.e., 2% relative to the final volume of polyol). This solution was then introduced into the polyol to achieve a final concentration of 10 mM. The resulting samples and their corresponding codes are detailed in Table 1. The obtained solution of polyol/Ag^2+^ was degassed with argon for 30 min at a low gas flow rate of 50 mL/min, then sealed in an airtight container before undergoing irradiation. Gamma irradiation took place in a laboratory irradiation facility (Ob-Servo Sanguis, Izotop, Budapest, Hungary) at room temperature with a dose rate of 0.5 kGy/h, resulting in a total integrated irradiation dose of 50 kGy.

After irradiation, the polyol/AgNPs solution was used to obtain PUF/AgNPs, using the recipe shown in Table 2.

### 2.3. Characterization of PUF/AgNPs

#### 2.3.1. Spectroscopic Analysis

UV–vis absorption spectroscopy (V-570 UV–vis spectrophotometer, Jasco, Hachioji, Japan) was used to determine the optical properties, to confirm the formation of AgNPs into the polyol, and to study the stability of both polyol/AgNPs and PUF/AgNPs. The analyses were performed in the wavelength range of 350–800 nm, using quartz cells with a 1 cm optical path and at a resolution of 1 nm (for liquid samples) and integrating spheres (for foams).

To study the gamma radiation influence on polyol’s and foams’ structure, infrared spectroscopy (FTIR-4200 spectrometer, Jasco, Hachioji, Japan) was used. The spectra were recorded using an ATR PRO470-H (Attenuated Toal Reflectance, JASCO) accessory in the spectral range of 4000–400 cm^−1^, at a resolution of 4 cm^−1^, and 200 scans for each spectrum.

#### 2.3.2. DSC Analysis

DSC measurements were performed with Setaram 131 EVO (Setaram Instrumentation, Caluire-et-Cuire, France) in the following conditions: temperature range: 30–300 °C; heating rate, 10 °C/min; atmosphere—air (gas flow: 50 mL/min). Samples of about 3–5 mg were placed in aluminum pans of 30 μL with pierced lids. The OOT (onset oxidation time) parameters were obtained from DSC curves using the Setaram Calisto software processing. Temperature calibration was checked in respect to indium standard.

#### 2.3.3. SEM-EDX Analysis

Evaluation of the appearance of both control and modified PU foams was performed with a scanning electron microscope TM4000plus II, SEM (Hitachi Ltd., Tokyo, Japan) equipped with an EDX accessory (Oxford AztecOne, Oxford Instruments, High Wycombe, UK). The electron accelerating voltage was 10 kV, and a standard vacuum was used in the sample chamber as specified by the manufacturer.

#### 2.3.4. Mechanical Properties

##### Dynamic Mechanical Analysis (DMA)

DMA analysis is a technique for measuring the mechanical properties of materials under the action of periodic mechanical stress, such as cyclic compressive force. The analysis was performed using a DMA Q800 mechanical analyzer (TA Instruments, New Castle, USA) on samples cut in the form of a parallelepiped with a base of 15 × 15 mm and a height of 10 mm at a temperature of 30 °C with a force variation of 0.5 N/min from 0 to 18 N.

##### Compression Resistance and Tensile Tests

The mechanical compression resistance of polyurethane foam using the classical method was determined using an LFM 30 kN mechanical testing machine (Walter & Sai AG, Tübingen, DE, USA). Test specimens were prepared with the following dimensions: length of 30 mm, width of 30 mm, and thickness of 4–7 mm. The tests were conducted according to ISO 844 Standard [26] in the parallel direction to the growth direction of the PU foam, with a force of 5 N and a compression speed of 5 mm/min. Using the same equipment, tensile tests were performed under the following conditions: test specimens were prepared with the following dimensions: length approximately 80–100 mm, width 5–8 mm, and thickness 5–8 mm. The tests were conducted according to ISO 1926 Standard [27] in the perpendicular direction to the growth direction of the PU foam, with a force of 10 N, a pulling speed of 10 mm/min. For each type of material, 3–4 trials were conducted, and subsequently, the average parameter values were calculated.

### 2.4. Measurement of Antimicrobial Activity

#### 2.4.1. Assessment of Antibacterial Activity

The antimicrobial activity of the PUF samples was evaluated against four standard strains: *Staphylococcus aureus* ATCC 25923, *Enterococcus faecalis* ATCC 29212, *Escherichia coli* ATCC 25922, and *Pseudomonas aeruginosa* ATCC 27853.

Determination of the PUF samples’ influence on bacterial viability was performed after the samples (previously sterilized by UV exposure for 30 min on each side) were put in contact with microbial suspensions in liquid medium. After different time intervals (6 h, 24 h, and 48 h), the microbial viability was established by making serial dilutions and seeding on agar medium in order to calculate the number of colony-forming units (CFU).

#### 2.4.2. Absorption Method (Direct Contact Test)

One of the most common methods for characterizing the quantitative antimicrobial effect of textile materials, including PUF, is the absorption method, which is presented in the JIS L1902:2015 standard [28]. This method is globally recognized and involves the assessment of the reduction of microorganisms compared to their initial concentrations and control samples (PUF without Ag NPs). The quantitative evaluation of polyurethane foam materials in this study was conducted after an incubation time of 24 h.

#### 2.4.3. Assessment of Fungicidal Activity

For evaluating the antifungal efficiency, samples of polyurethane foam (20 × 20 × 3 mm) were placed in Petri dishes containing a medium suitable to the growth of fungal strains, i.e., Chapex–Dox agar, following standard G21 [29]. A fungal spore suspension, consisting of a mixture of *Aspergillus brasiliensis*—ATCC 9642, *Penicillium funiculosum*—ATCC 11797, *Chaetomium globosum*—ATCC 6205, *Trichoderma virens*—ATCC 9645, and *Aureobasidium pullulans* ATCC 15233, was sprayed on both the sample surfaces and the medium surface. Subsequently, the samples were incubated for 28 days at 28 ± 2 °C and a relative air humidity of 95%.

### 2.5. In Vitro Biocompatibility Assessment

#### 2.5.1. Cell Culture

The human HaCaT keratinocytes cell line was purchased from Cell Lines Service (CLS) (catalogue number 300493, Eppelheim, Germany). The cells were cultured in Dulbecco’s Modified Eagle Medium (DMEM; Gibco/Invitrogen, Carlsbad, CA, USA) with an addition of 10% fetal bovine serum (FBS; Gibco/Invitrogen, Carlsbad, CA, USA) at 37 °C in a humidified atmosphere with 5% CO_2_. Polyurethane foams (PUF) were tested for cytotoxic effects according to a modified version of ISO 10993-5 “Biological evaluation of medical devices—Part 5: Tests for in vitro cytotoxicity”, using conditioned medium (PUF extract in cell growth medium) [30]. Thus, PUF extracts were prepared by incubating approximately 1.5 mg of each test sample in 1.5 mL of complete DMEM medium for a period of 24 h at 37 °C. Epithelial cells were seeded in 96-well plates at a density of 2 × 10 ^4^ cells/well and allowed to adhere overnight. Subsequently, the medium was removed and replaced with 200 μL of extract previously sterilized by UV exposure. After 24 h of cell exposure, several biocompatibility tests were performed.

#### 2.5.2. MTT Assay

The cell viability was measured using the 3-(4,5-dimethylthiazol-2-yl)-2,5-diphenyltetrazolium bromide (MTT) assay, which is based on the quantification of NAD(P)H-dependent cellular oxido-reductase enzymes activity in the viable cells. After 24 h of cell exposure, PUF extracts were removed, and the keratinocytes were subsequently incubated with 1 mg/mL MTT solution for 2 h at 37 °C. The purple formazan crystals formed in the viable cells were dissolved with 200 μL of isopropyl alcohol, and the absorbance was measured at 595 nm using a FlexStation 3 microplate reader (Molecular Devices, San Jose, CA, USA).

#### 2.5.3. Determination of Nitric Oxide (NO) Level

The level of NO released in the culture medium as an indicator of inflammation was determined using the Griess reagent. Culture supernatants were mixed with an equal volume of Griess reagent, which is a stoichiometric solution (*v*/*v*) of 0.1% naphthylethylenediamine dihydrochloride and 1% sulfanilamide in 5% H_3_PO_4_, and absorbance was read at 550 nm using a FlexStation 3 microplate reader (Molecular Devices, San Jose, CA, USA).

#### 2.5.4. Cytotoxicity Test Based on Lactate Dehydrogenase Activity

The destruction of the cell membrane leads to the release of intracellular lactate dehydrogenase (LDH) into the culture medium, which can be quantified by a series of coupled reactions that ultimately lead to reduction of a tetrazolium salt to a red formazan, which can be measured spectrophotometrically. The LDH amount released in culture medium was determined as a measure of cell membrane integrity and cell viability using a commercial kit (Cytotoxicity Detection Kit-LDH, Roche, Basel, Switzerland). Briefly, volumes of 50 μL of culture supernatants were incubated with a 50 μL assay reagent for 30 min in dark. The absorbance was then measured at 490 nm using a FlexStation 3 microplate reader (Molecular Devices, San Jose, CA, USA).

#### 2.5.5. Determination of the Intracellular Level of Reactive Oxygen Species (ROS)

The intracellular level of ROS was assessed using a fluorescent compound 2′,7′-dichlorofluorescein diacetate (DCFH-DA). Keratinocytes were washed with PBS and incubated with the dye for 30 min at 37 °C. Subsequently, excess dye was removed, and the fluorescence was quantified using a FlexStation 3 microplate reader (Molecular Devices, San Jose, CA, USA) (wavelength of excitation and emission set at 488 nm and 515 nm, respectively). All results were expressed relative to control after fluorescence intensity was reported to the number of viable cells in each sample.

#### 2.5.6. Filamentous Actin and Nuclei Staining

The cell cytoskeleton morphology was visualized via fluorescence microscopy using cells fixed with 4% paraformaldehyde for 20 min and permeabilized with 0.1% Triton X-100—2% bovine serum albumin for 1 h. Filamentous actin (F-actin) was labeled with 20 μg/mL phalloidin conjugated with fluorescein isothiocyanate (FITC) (Sigma-Aldrich, Munich, Germany), and nuclei were counterstained with 2 μg/mL 4′,6-diamidino-2-phenylindole (DAPI) (Molecular Probes, Life Technologies, Carlsbad, CA, USA). Images were captured using a fluorescence microscope Olympus IX71 (Olympus, Tokyo, Japan).

### 2.6. Statistical Analysis

The in vitro assays were performed in triplicate, and the results were presented as mean ± standard deviation (SD) of three independent experiments. The statistical significance was analyzed by Student’s *t*-test. A value of *p* less than 0.05 was considered significant.

## 3. Results and Discussion

### 3.1. Radiochemical Synthesis of PUF Nanocomposites

Ionizing radiation represents a commonly used method for modifying materials, aiming to enhance certain characteristics such as mechanical, electrical properties, degradation stability, etc., as well as for obtaining new materials, including metallic nanoparticles. Radiochemical synthesis of metal nanoparticles in aqueous solutions stands out as an environmentally sustainable and industrially viable method for swiftly synthesizing metal nanoparticles with precise control over crucial properties such as shape, size, and homogenous dispersion [21,22,31,32]. This approach offers significant advantages in terms of efficiency and environmental impact, as it minimizes the need for harsh chemicals and reduces waste generation compared to conventional synthesis methods [31,32].

**Figure 1:**
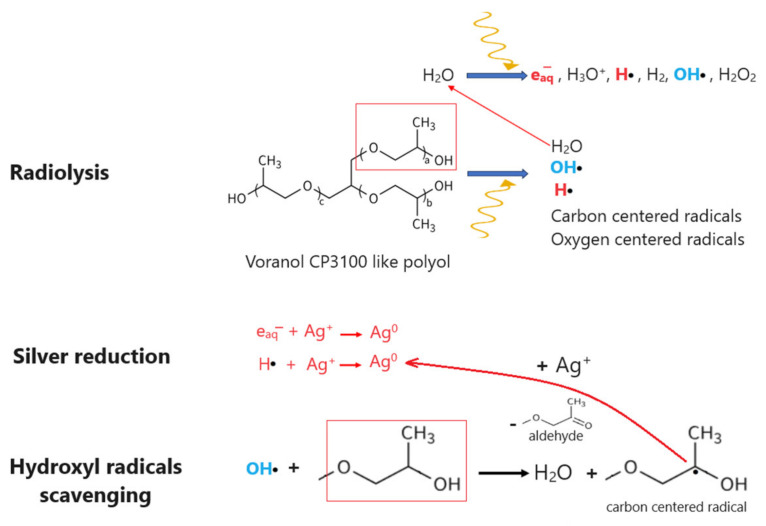
Radiochemical synthesis of polyol/AgNPs.

In the radiochemical synthesis process of silver nanoparticles, a polyol/H_2_O/Ag^+^ solution undergoes gamma irradiation. The gamma radiation energy causes ionization and excitation processes within both the water and the polyol matrix, leading to the formation of highly reactive species (Figure 1), including hydrated electrons (e_aq_^−^) and hydrogen radicals (H●), which possess strong reducing capabilities [33]. As a consequence of these reactive species, silver ions (Ag^+^) within the solution are efficiently reduced to silver atoms (Ag^0^), facilitating the nucleation and growth of silver nanoparticles. The radiolysis of polyols could also lead to the formation of various carbon and oxygen centered radicals, hydroxyl radicals, and water [34,35].

Furthermore, the radiolysis process generates hydroxyl radicals (OH●), known for their strong oxidizing properties [33]. These radicals have the potential to oxidize silver nanoparticles (AgNPs); thus, it becomes imperative to mitigate their presence in the reaction medium. Typically, primary or secondary alcohols are employed for this purpose, serving to neutralize and scavenge these highly reactive hydroxyl radicals [21,31,36]. In our case, the polyol matrix can directly capture hydroxyl radicals, resulting in the formation of water and a carbon-centered radical. This carbon-centered radical can then actively participate in the reduction of silver ions, leading to the formation of aldehyde-type compounds [37]. This proposed mechanism not only helps in neutralizing the oxidizing hydroxyl radicals but also contributes to the reduction process of silver ions, ultimately aiding in the synthesis of silver nanoparticles within the polyol solution.

**Figure 2:**
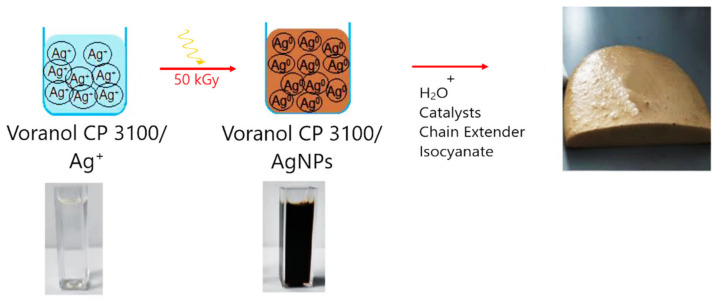
Steps for obtaining of PUF/AgNPs.

After irradiation, a notable color change occurred in the polyol solution, transitioning from a transparent appearance to a deep, dark brown color, serving as an initial indication of silver nanoparticle formation within the polyol (Figure 2). The polyurethane foam was synthesized using the conventional method for polyurethane foam production, following the recipe outlined in Table 2. The hydroxyl (-OH) groups of the polyol/AgNPs react with the isocyanate (-NCO) groups of the diisocyanate, leading to the formation of urethane linkages (-NHCOO-) and the release of carbon dioxide (CO_2_) gas as a byproduct [38,39,40]. Water can also react with isocyanate groups to form urea linkages (-NHCONH-) and generate CO_2_. The CO_2_ gas creates bubbles within the polyurethane matrix, causing it to expand and form a foam structure [38]. Both crosslinking and gas generation reactions are catalyzed by the presence of tertiary amines (e.g., Dabco 33-LV) and tin salt (e.g., Dibutyltin dilaurate). Surfactants (e.g., Vorasurf DC 2585) aid in bubble stabilization and size control by reducing surface tension at the gas–liquid interface [38,39,41]. The extent of crosslinking and the final foam density can be controlled by adjusting the ratio of reactants, the choice of catalysts, and the reaction conditions [38,39].

We aimed to explore the influence of certain compounds renowned for their ability to stabilize metal nanoparticles (e.g., PVP—V2 and SDS—V3) [42,43] as well as those with hydroxyl radicals scavenging capabilities (e.g., BDO—V4) on both the stability of silver nanoparticles and the final characteristics of polyurethane foam. Thus, samples containing polyol/H_2_O/Ag^+^/PVP, SDS, or BDO were subjected to irradiation under identical conditions as polyol/H_2_O/Ag^+^.

### 3.2. Characterization of Polyol and PUF Nanocomposites

#### 3.2.1. Stability of Nanoparticle Systems

Silver nanoparticles exhibit a characteristic surface plasmon resonance (SPR) detectable by UV–vis spectroscopy [21,44]. This technique provides insights into their optical properties by monitoring the SPR peak’s position over time, and the stability of silver nanoparticles or nanocomposites (such as PUF/AgNPs) can be assessed. A lack of significant SPR shifts indicates a highly stable system.

**Figure 3:**
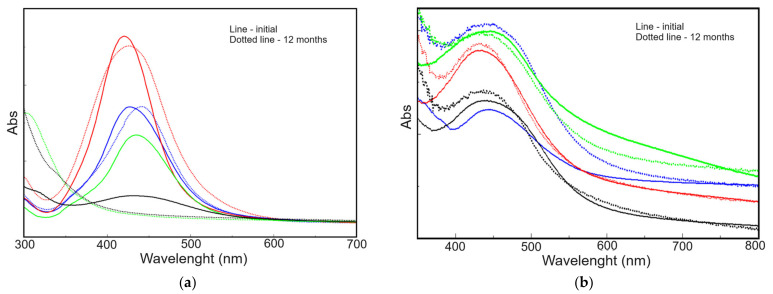
UV–vis spectra recorded on: (**a**) polyol/AgNPs and (**b**) PUF/AgNPs: V1—blue, V2—red, V3—black, and V4—green.

For assessing the long-term stability of both obtained nanoparticle systems, namely polyol and polyurethane foam, UV–vis measurements were performed on samples at the initial time point (immediately after radiochemical synthesis) and after 12 months (Figure 3a,b).

UV–vis analysis revealed instability in polyol/AgNPs samples stabilized with BDO (V3) and SDS (V4), as their SPR peak disappeared after 12 months. In contrast, PUF/AgNPs maintained their SPR peaks, suggesting good stability of the metallic nanoparticles within the foam structure. Interestingly, sample V1 showed a slight increase in SPR peak intensity over time. This might be due to nanoparticle rearrangement or migration towards the sample surface (where the analysis was performed), potentially caused by reduced nanoparticle mobility due to stabilizing agents in other samples. This aspect correlates, as we will see in the corresponding section, with antimicrobial activity data, where sample V1 was generally the most effective against all types of tested microorganisms.

#### 3.2.2. ATR-FTIR Analysis

**Figure 4:**
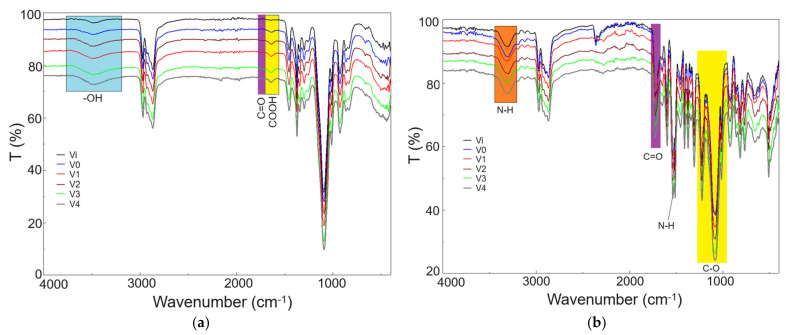
FTIR spectra recorded on: (**a**) polyols and (**b**) PUF.

FTIR analysis was employed to observe structural modifications resulting from both irradiation and the introduction of silver nanoparticles into the foam structure. The FTIR spectra recorded on polyol and polyurethane foam samples are presented in Figure 4a and Figure 4b, respectively. In the case of polyol samples (Figure 4a), the FTIR spectra exhibit slight changes in regions corresponding to hydroxyl groups (-OH, 3300–3500 cm^−1^), carbonyl (C=O, 1710–1730 cm^−1^), and carboxyl (COOH, 1500–1700 cm^−1^) [36,45], with their intensity increasing from V0 to V4. However, comparing sample V0 (50 kGy, without AgNPs) to sample V1 (50 kGy, with AgNPs), the spectra are similar, except for the disappearance of the peak at 1723 cm^−1^ (C=O) in sample V1, possibly due to AgNPs attachment. The FTIR spectra recorded on PUF foams (Figure 4b) exhibit characteristic bands of polyurethane foams [46,47], including N-H (3210–3430 cm^−1^), C=O (1670–1760 cm^−1^), and C-O (980–1260 cm^−1^). Assessing the influence of AgNPs and polyol irradiation on the chemical structure of PUF via FTIR analysis is challenging due to the complex spectrum structure and the overlapping bands specific to AgNPs interaction with the polyurethane matrix.

#### 3.2.3. DSC Analysis

Differential scanning calorimetry (DSC) analysis is a powerful technique used in the investigation of thermal properties and transitions in various materials, including polyols and polyurethane foam. By subjecting samples to controlled heating cycles, DSC provides valuable insights into phase transitions and thermal stability of tested materials [48].

**Figure 5:**
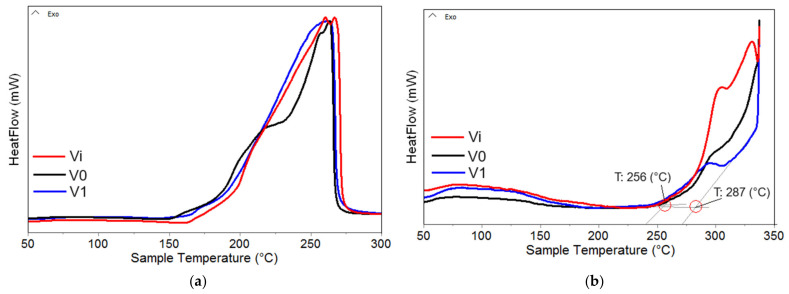
DSC curves recorded on: (**a**) polyols and (**b**) PUF.

Figure 5a,b display the DSC curves obtained under an oxidizing atmosphere for polyol and the corresponding PUF/AgNPs samples. These curves were used to determine the onset oxidation temperature (OOT) values [49], as outlined in Table 3, which serve as an indicator of the materials’ stability. OOT values, defined as the temperature at which oxidation reactions commence, are directly associated with the resistance to thermo-oxidation exhibited by the tested materials.

Analysis of the DSC data reveals several key observations: (i) Polyols exhibit significantly lower oxidation stability compared to their corresponding foams; (ii) the irradiation process induces a slight decrease in onset oxidation temperature (OOT) values (observed in Vi vs. V0), attributed to the generation of radio-induced free radicals that, in combination with oxygen, form various oxidation intermediate compounds, initiating a chain reaction until the deactivation of these radicals through recombination reactions or interaction with antioxidant compounds [48,49]; (iii) the presence of AgNPs appears to exert an antioxidant effect, potentially due to their electronic properties, which deactivate some of the free radicals generated during irradiation, resulting in OOT values (especially in polyols) close to those of non-irradiated samples [50]; and (iv) DSC oxidation curves of polyurethane foams exhibit a two-stage structure, indicating an initial oxidation process (OOT_1_) followed by a thermal combustion/decomposition process (OOT_2_) of the samples at high temperatures. These insights provide valuable understanding of the oxidative behavior and stability of the materials, facilitating optimization and improvement for various applications.

#### 3.2.4. SEM-EDX Analysis

**Figure 6:**
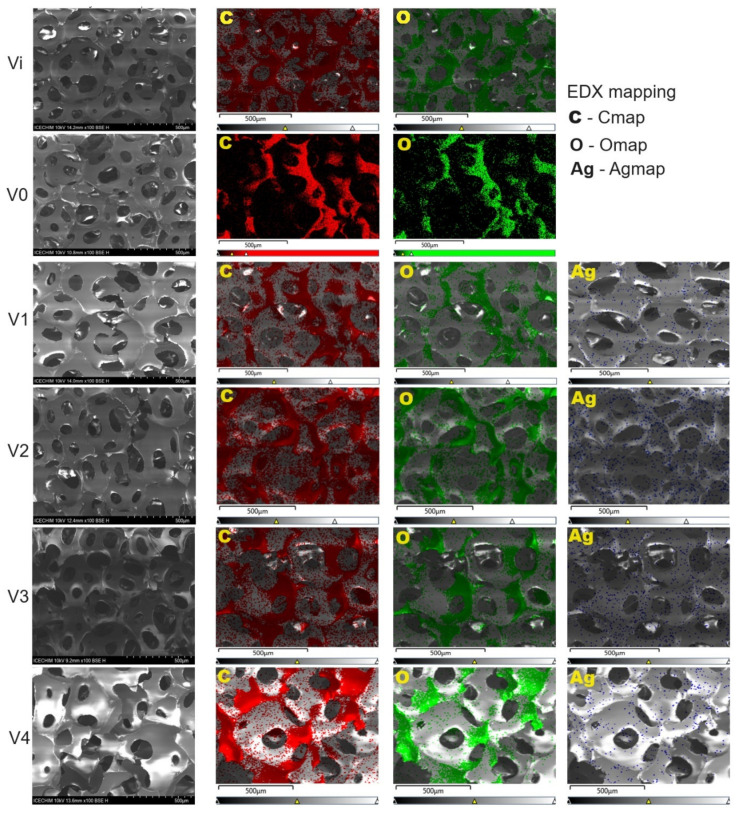
SEM-EDX analysis results.

The morphostructural characterization of PUF/AgNPs samples was performed using SEM-EDX analysis (Figure 6). Overall, the SEM analysis indicates that there are generally no significant structural differences between PUF/AgNPs foams (V1, V2, and V3) and those without Ag (Vi and V0), with all polyurethane foams presenting an open cell structure with a cell window ranging between 50–150 µm [51,52]. The average size of open cells appears to decrease upon the inclusion of silver nanoparticles. As underlined by studies in the literature [53], the addition of fillers can lead to visible damages of the cell structure as well as to the induction of non-uniformity in cell morphology. However, in the case of sample V4, the formation of distinct crystalline deposits on the sample’s outer surface is observed. These deposits are likely sodium dodecyl sulfate (SDS) that has migrated and crystallized. This phenomenon could be potentially attributed to incompatibility between SDS and the polyurethane foam matrix.

In the same time, from the SEM-EDX analyses can be noticed the preservation of the smooth skeletons of the unmodified foam, without the presence of rough surfaces, as noticed by other authors upon modification of PU foams with titania nanoparticles [54].

The EDX analysis conducted on these samples correlates very well with the UV–vis data, wherein stability tests showed that systems V3 and V4 are more unstable, meaning that the AgNP concentration decreases. Thus, the EDX data (Figure S1a–d) confirmed the presence of Ag nanoparticles in the structure of V1–V4 foams, with silver concentrations as follows: 0.4 wt% (V1), 0.3 wt% (V2), and 0.2 wt% (V3 and V4), respectively.

#### 3.2.5. Mechanical Properties

**Figure 7:**
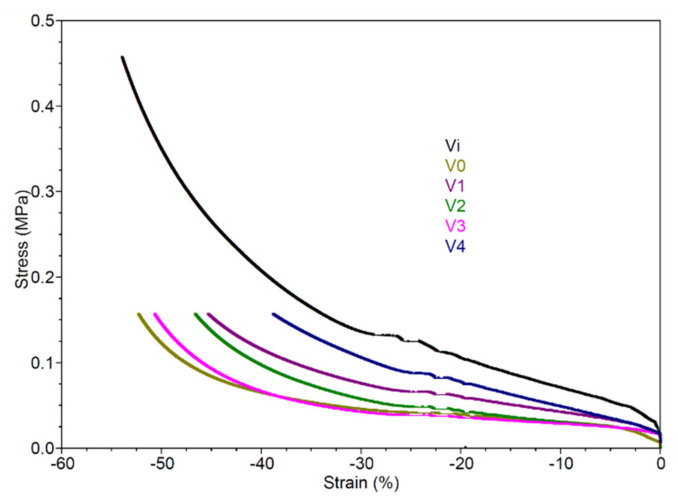
DMA analysis: Stress vs. Strain.

In Figure 7, the *stress* vs. *strain* graph illustrates that sample Vi experiences a deformation of 53.16% at 18 N, reaching a stress of 0.45 mPa. With exposure to irradiation and the inclusion of Ag nanoparticles, under a stress of approx. 0.16 mPa (18 N force), deformations for V0, V1, V2, V3, and V4 are 52.35%, 50.64%, 46.69%, 45.37%, and 38.70%, respectively. It is evident that lower deformations correlate with a more stable structure of the tested material [55], implying that the incorporation of Ag nanoparticles serves to stabilize the polyurethane foam structure.

**Figure 8:**
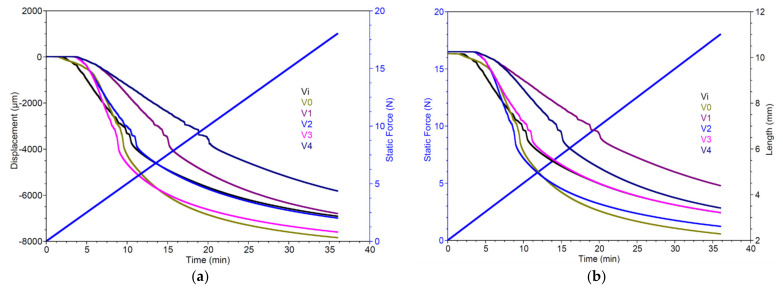
DMA analysis: displacement vs. time (**a**) and length vs. time (**b**).

In the DMA displacement vs. time graphs (Figure 8), sample V1 displays a notably higher level of hardness compared to samples Vi and V0, which is evident from the deformation measurements of 1682 µm (V1), 3387 µm (Vi), and 4307 µm (V0) under a 5 N force. Additionally, sample V3 behaves similarly to V0, exhibiting a minor increase in stiffness when subjected to forces exceeding 4 N. Notably, samples V1, V2, and V4 demonstrate zero deformation when forces up to 2.5 N are applied. Under higher forces (10–18 N), a decelerated deformation is apparent for V4, followed by sample V1, which exhibits marginally greater deformation than the reference sample Vi.

The mechanical properties of polyurethane (PU) foams are intricately linked to their microstructural parameters, including cell size and relative density, as noted in previous studies [56]. Moreover, several researchers have studied the impact of incorporating silver nanoparticles into PU formulations, revealing a notable enhancement in mechanical performance [57,58,59].

**Figure 9:**
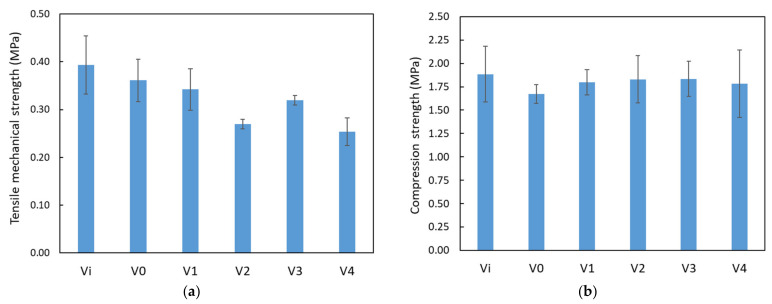
Mechanical resistance of PUF samples to (**a**) compression and (**b**) tensile tests.

Similar to the findings in the DMA analysis, classical compression and tensile tests revealed a parallel trend (Figure 9). Notably, samples treated with Ag nanoparticles exhibited heightened compression strength in comparison to their untreated counterparts. In the context of tensile testing, it is worth mentioning that sample V1 demonstrated the highest tensile strength among those treated with Ag nanoparticles, with samples V3, V2, and V4 following in sequence. This observation emphasizes the strengthening impact of Ag nanoparticles on the mechanical characteristics of the tested material in both compression and tension, thereby accentuating their potential to enhance the structural integrity and performance of PUF nanocomposites.

#### 3.2.6. Antimicrobial Properties

The antimicrobial activity assessment of PUF nanocomposites encompassed various microbiological test methodologies, including bacteria viability testing, absorption method (direct contact test), and exposure to mixed fungal suspensions.

**Figure 10:**
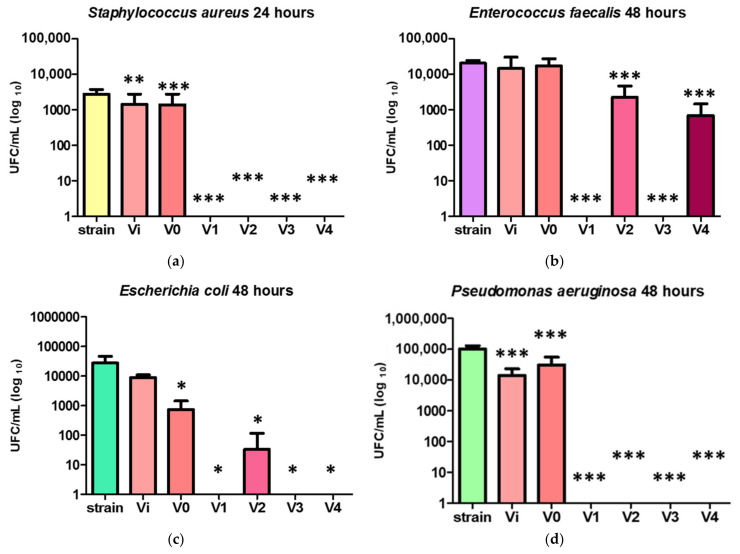
Assessment of microbial viability of *Staphylococcus aureus* (**a**), *Enterococcus faecalis* (**b**), *Escherichia coli* (**c**), and *Pseudomonas aeruginosa* (**d**) in the presence of PUF/AgNPs samples. Results were calculated as the mean ± standard deviation (SD) (n = 6). * *p* < 0.05, ** *p* < 0.01, and *** *p* < 0.001 compared to strain without material.

The impact of PUF on microbial growth was not uniform and varied depending on several factors. These included the type of bacteria strains and the exposure time. Different microorganisms exhibited different sensitivities to the properties of the foams, including their surface chemistry. Moreover, prolonged exposure increased the likelihood of the foams to disrupt microbial membranes upon contact, releasing inhibitory ions or even trapping microbes within its porous structure, resulting in a gradual decline in growth over time (Figure 10).

The analysis of the impact of PUF on bacterial growth revealed several key findings. All PUF materials containing Ag nanoparticles started to inhibit the growth of *S. aureus* strain after just 6 h of contact (Figure S2), leading to the absence of viable cells after 24 h (Figure 10a). For *E. faecalis* (Figure 10b), the strongest inhibitory effect was observed in the case of V1 and V3 samples, where no viable bacterial cells were recovered after 48 h. Additionally, samples V2 and V4 exhibited a significant reduction in the number of viable cells compared to control. Concerning the Gram-negative strains (*E. coli* and *P. aeruginosa)* (Figure 10c,d), it was noted that materials containing silver nanoparticles hindered bacterial growth after a minimum contact period of 6 h (Figure S2), and no viable bacteria were recovered after 48 h of incubation with V1, V3, and V4, with low numbers of *E. coli* being detected solely in the case of sample V2.

Our results confirmed that the introduction of nanosilver into PUF systems increased their antimicrobial properties, most probably due to the continuous release of silver ions. The great antibacterial efficiency against both Gram-negative (*E. coli* and *P. aeruginosa*) and Gram-positive (*S. aureus* and *E. faecalis*) bacteria has been previously reported in Ag-modified PU matrices (Table 4) [60,61,62].

**Figure 11:**
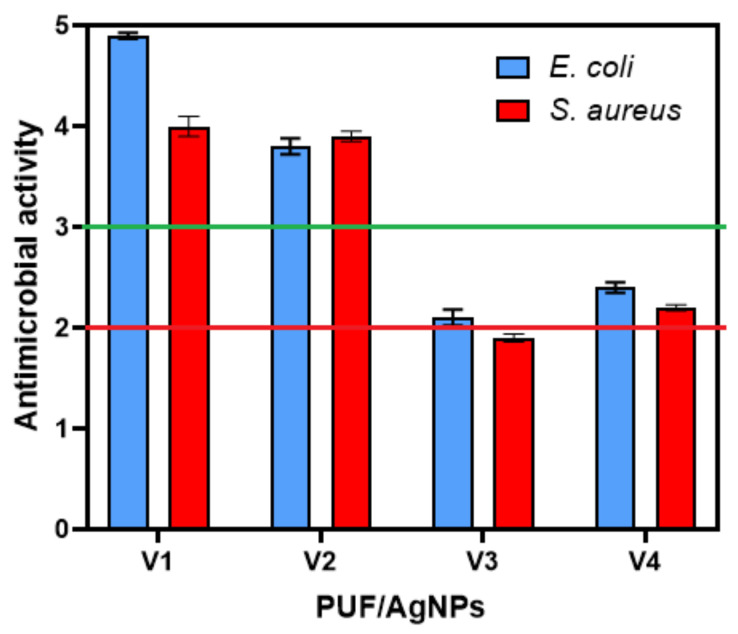
Antimicrobial activity determined by absorption method [28]. The dashed lines represent the two lower limits of antimicrobial activity for the tested material to be considered as having antimicrobial effect (red line) and full antimicrobial effect (green line).

Figure 11 illustrates the antibacterial efficacy of polyurethane foam materials incorporated with silver nanoparticles when exposed to two bacterial strains, namely *S. aureus* and *E. coli*, utilizing the adsorption method [28].

What is specific to this standard [28] is that serial dilutions to quantify the number of microbial colonies are performed in a nutrient medium (this allows microorganisms to grow even during the test), whereas in other microbiological methods, dilution is carried out with sterile deionized water (non-nutrient medium). For a material to be considered to have antimicrobial effect, it must have an antimicrobial activity value (A) of 2 ≤ A < 3, and to be considered (“*Full*”), it must be A > 3 [28]. It is evident from the results that samples V1 and V2 exhibit complete antimicrobial activity against both bacterial strains used, while samples V3 and V4 demonstrate a partial antimicrobial effect.

In Table 5, the results obtained from testing the effectiveness of PUF/NPAg exposure to various fungal strains are presented, conducted in accordance with the ASTM G21 standard [29]. According to the ASTM G21 standard, after 28 days of incubation, the degree of fungal growth on the sample surface can be evaluated based on the coverage level, rated on a scale from 0–4, where a value of 0 indicates no growth, and 4 indicates strong growth covering the entire sample surface. Materials with scores of 0–1 are considered resistant to fungal action, while materials scoring 2–4 are deemed non-resistant to fungal action.

Exposure to a mixture of fungi revealed that all polyurethane foams containing silver nanoparticles exhibited high efficacy against the development of the tested fungi.

#### 3.2.7. Cytotoxic Effects of PUF/NPAg on Human Keratinocytes

Polyurethane foams possess unique properties that make them well-suited for biomedical applications. Their porous structure, permeability, and high surface area-to-volume ratio enable efficient fluid absorption, gas exchange, and tissue integration. Additionally, the mechanical properties of polyurethane foams can be tailored to match specific application requirements, allowing for the creation of scaffolds and implants with desired strength, elasticity, and biodegradability. This customizability facilitates the development of patient-specific solutions, contributing to improved treatment outcomes. From wound dressings to tissue engineering scaffolds, drug delivery systems, and medical implants, polyurethane foams have demonstrated their potential in advancing healthcare [8,63].

However, concerns have been raised regarding the cytotoxicity of polyurethane foams, specifically their potential impact on human health. When evaluating the cytotoxicity of polyurethane foams, researchers focus on their potential to induce adverse effects on living organisms, especially on human cells. The complex composition of polyurethane foams, which typically includes isocyanates, polyols, and various additives, may contribute to their cytotoxic properties [64].

The potential cytotoxic effects of silver nanoparticles containing PUFs were evaluated by various in vitro tests performed on normal human keratinocytes, targeting cell proliferation capacity and membrane integrity as well as their potential to generate an inflammatory response and oxidative stress.

**Figure 12:**
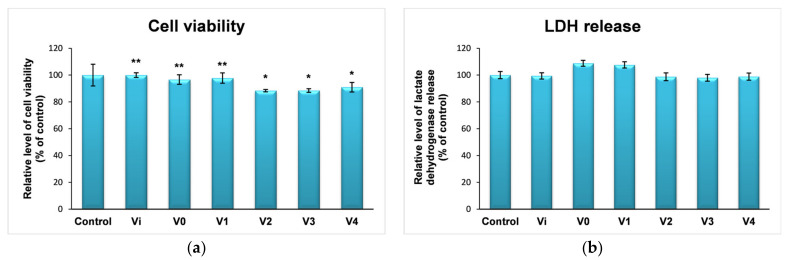

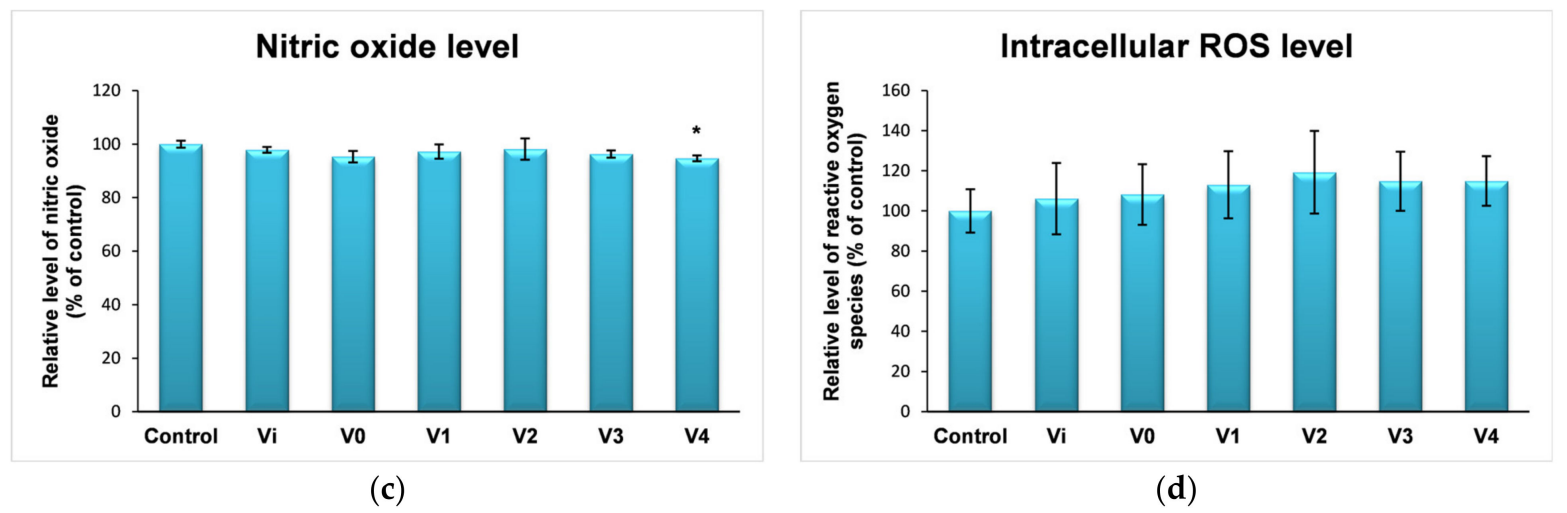
Cellular response revealed by the cell viability (**a**), LDH (**b**), and NO (**c**) release as well as intracellular ROS level (**d**) after 24 h exposure of human keratinocytes to PUF-derived conditioned media. Results were calculated as the mean ± standard deviation (SD) (n = 3) and represented relative to the control (untreated cells). * *p* < 0.05 and ** *p* < 0.01 compared to control.

The results of the MTT test (Figure 12a) showed that the incubation of human keratinocytes with PUF-derived conditioned media did not significantly influence cell viability; they caused a decrease in the number of cells by only 10% after 24 h of exposure to the foams obtained with different stabilizing agents. These results were consistent with the level of LDH released into the culture media (Figure 12b). Thus, human keratinocytes preserved their membrane integrity after 24 h of exposure to PUF. In addition, the nitric oxide level did not undergo significant changes (Figure 12c), indicating that the short-term (24 h) exposure of the epithelial cells to the analyzed PUFs did not generate inflammatory responses. Also, exposure of human keratinocytes to PUF-derived conditioned media generated a non-significant increase in the level of ROS, the maximum value (20% compared to control) being recorded in the case of foam obtained with SDS (Figure 12d).

**Figure 13:**
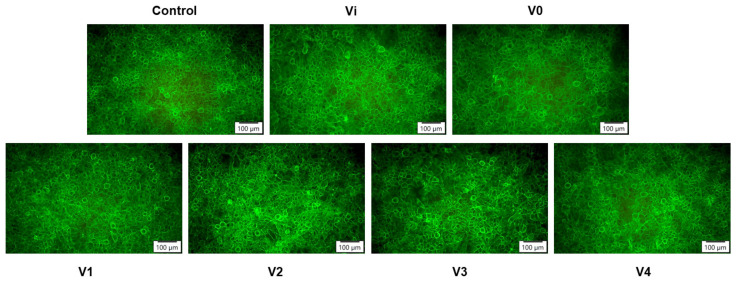
Representative fluorescence images showing cytoskeletal actin filaments in human keratinocytes after 24 h of exposure to PUF derived conditioned media. F-actin (green) staining was done by phalloidin-fluorescein isothiocyanate (FITC). Scale bar: 100 µm.

The actin cytoskeleton organization revealed by fluorescence microscopy in Figure 13 was consistent with the results of the biocompatibility tests previously presented. Thus, it could be observed that the cells kept their specific morphology after 24 h of incubation, which confirmed that the tested PUF extracts did not affect the behavior of human keratinocytes, proving good biocompatibility.

In comparison with our results, there are also previous reports on biocompatibility and wound-healing properties of PUF modified with various types of nanoparticles (Table 6).

## 4. Conclusions

The radiochemical in situ synthesis of silver nanoparticles within polyol to produce polyurethane foam nano-composites offers a pioneering approach with substantial potential for advancing new materials featuring polyurethane/metallic nanoparticle blends. This method facilitates the direct integration of silver nanoparticles into the polyol matrix, eliminating the need for subsequent addition during polyurethane foam manufacturing, unlike traditional methods.

The results obtained from the characterization of polyurethane foams with silver nanoparticles led to a relevant and useful conclusion regarding the improvement of these materials’ properties: The addition of an additional stabilizing agent is not necessary. This is because the chemical structure of the polyol, with the presence of hydroxyl groups, is sufficient to ensure the efficient stabilization of silver nanoparticles.

The PUF/AgNPs samples were harmless to human keratinocytes in the case of a short-term exposure. Our results showed that none of the tested samples induced significant changes in the viability of HaCaT cells, did not cause damage to the structure of plasma membranes, and did not exert pro-inflammatory effects. At the same time, there were no significant increases in the intracellular ROS level.

With the advantage of achieving a uniform distribution of nanoparticles, the resulting nanocomposites can yield enhanced properties such as increased mechanical strength and antimicrobial properties for biomedical applications such as hospital bed mattresses.

## Figures and Tables

**Table 1:** Encoding of the polyol/AgNPs samples.

Code	[Ag^+^] (mM)	Dose (kGy)	Stabilizing Agent (2%)	Hydroxyl Radical’s Scavenger (2%)
Vi	0	0	-	-
V0	0	50	-	-
V1	10		-	-
V2			PVP	-
V3			-	BDO
V4			SDS	-

**Table 2:** Protocol for synthesizing PUF/AgNPs.

Compound	Parts
Polyol/AgNPs	100
BDO	7.5
Water	3.0
Dabco 33-LV	0.4
Dibutyltin dilaurate	0.24
Vorasurf DC 2585	1.0
4,4′-Methylene diphenyl diisocyanate prepolymer (ISO 137/28)	200.0

**Table 3:** OOT values recorded on polyol and polyurethane foams.

Sample	OOT (°C)		
	Polyol (Liquid)	PUF	
		OOT_1_ (°C)	OOT_2_ (°C)
Vi	188	281	312
V0	180	274	308
V1	185	258	292
V2	178	257	284
V3	189	252	301
V4	184	250	292

**Table 4:** Antibacterial effects of our PU matrix compared to other silver-nanoparticle-modified PU foams.

PU Matrix	Main Findings on Antibacterial Activity	Ref.
Polyurethane foam embedded with silver nanoparticles (PUF/AgNPs)	Microbicidal activity against *S. aureus* strain at 24 h incubation time Microbicidal activity against *E. coli*, *E. faecalis*, and *P. aeruginosa* bacteria after 48 h treatment with V1, V3, and V4 samples	Our study
Pristine PUFs with AgNPs	Microbicidal activity against *E. coli* bacteria at 6.5 h incubation time	[60]
PUF with AgNPs and recombinant human epidermal growth factor (rhEGF)	Growth inhibition of *E. coli* and *S. aureus* treated with AgNP-PUFs and AgNP/rhEGF-PUFs after 24 h	[61]
PUF containing silver, alginate, and asiaticoside	Growth inhibition of *S. aureus*, *B. subtilis*, *E. coli*, and *P. aeruginosa* treated for 24 h	[62]

**Table 5:** Testing of samples by exposure to a fungal mix (incubation time: 28 days).

Sample	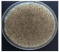	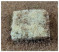	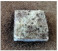	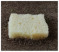	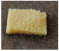	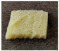	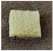
	Control	Vi	V0	V1	V2	V3	V4
Score according to [29]	4	4	4	0	0	0	0

**Table 6:** Biocompatibility and cytotoxic effects of our PU matrix compared to other silver-nanoparticle-modified PU foams.

PU Matrix	Main Findings on Biocompatibility	Ref.
Polyurethane foam embedded with silver nanoparticles (PUF/AgNPs)	PUF/AgNPs samples were harmless to human keratinocytes in the case of a short-term exposure	This study
PUF containing silver, alginate, and asiaticoside	Non-cytotoxic effects to human skin fibroblasts	[62]
Three-dimensional porous foam dressing with silver nanowires	High cell viability after 48 h of incubation with 3T3 cells and wound-healing promotion in pigs by combining with exogenous electric fields	[65]
Silver-modified PU foams	Good cytocompatibility with the 3T3 murine fibroblast line	[66]
PU foams with incorporated silver nanoparticles and recombinant human epidermal growth factor	Good cytocompatibility with mice fibroblasts L929 and significantly accelerated the healing of diabetic wounds, with complete re-epithelialization in a diabetic BALB/c mice mode	[61]
PU foams with incorporated lignin-capped silver nanoparticles	No cytotoxicity to HaCaT cells and BJ5ta fibroblasts; radical-scavenging activity and an ability to reduce the ex vivo myeloperoxidase activity in wound exudate	[67]

## Data Availability

Data are contained within the article and the Supplementary Materials.

## Supplementary Materials

The following supporting information can be downloaded at: https://www.mdpi.com/article/10.3390/polym16101369/s1, Figure S1: EDX data obtained for samples V1 (a), V2 (b), V3 (c), and V4 (d); Figure S2: Viability of *Staphylococcus aureus* (a), *Enterococcus faecalis* (b), *Escherichia coli* (c), and *Pseudomonas aeruginosa* (d) in the presence of PUF/AgNPs materials. Results were calculated as the mean ± standard deviation (SD) (n = 6). * *p* < 0.05, ** *p* < 0.01, and ** *p* < 0.001 compared to strain without material.
