# Supplementary material for: Gamma Radiation-Mediated Synthesis of Antimicrobial Polyurethane Foam/Silver Nanoparticles
Source: Polymers (Basel). 2024 May 10;16(10):1369. doi: 10.3390/polym16101369 (PMC11125184; doi:10.3390/polym16101369)
Supplement: Supplementary file 1 [file polymers-16-01369-s001.zip › polymers-2985518-supplementary.pdf]

SUPPLEMENTARY MATERIAL

Article

Gamma Radiation-Mediated Synthesis of Antimicrobial Polyurethane Foam/Silver Nanoparticles

Eduard-Marius Lungulescu [1], Radu Claudiu Fierascu [2,3], Miruna S. Stan [4], Irina Fierascu [2,5], Elena Andreea Radoi [1], Cristina Antonela Banciu [1], Raluca Augusta Gabor [2], Toma Fistos [2,3], Luminita Marutescu [6], Marcela Popa [6], Ionela C. Voinea [4,*], Sorina N. Voicu [4] and Nicoleta-Oana Nicula [1,*]

[1] National Institute for Research and Development in Electrical Engineering ICPE-CA, 313 Splaiul Unirii, 030138 Bucharest, Romania; marius.lungulescu@icpe-ca.ro (E.-M.L.); elena.radoi@icpe-ca.ro (E.A.R.); cristina.banciu@icpe-ca.ro (C.A.B.)

[2] National Institute for Research & Development in Chemistry and Petrochemistry—ICECHIM, 202 Spl. Independentei, 060021 Bucharest, Romania; fierascu.radu@icechim.ro (R.C.F.); irina.fierascu@icechim.ro (I.F.); raluca.gabor@icechim.ro (R.A.G.); toma.fistos@icechim.ro (T.F.)

[3] Faculty of Chemical Engineering and Biotechnology, National University of Science and Technology Politehnica Bucharest, 1-7 Gh. Polizu Str., 011061 Bucharest, Romania

[4] Department of Biochemistry and Molecular Biology, Faculty of Biology, University of Bucharest, 91-95 Splaiul Independentei, 050095 Bucharest, Romania; miruna.stan@bio.unibuc.ro (M.S.S.); sorina.voicu@bio.unibuc.ro (S.N.V.)

[5] Faculty of Horticulture, University of Agronomic Sciences and Veterinary Medicine of Bucharest, 59 Marasti Blvd., 011464 Bucharest, Romania

[6] Department of Microbiology, Faculty of Biology, University of Bucharest, 91-95 Splaiul Independentei, 050095 Bucharest, Romania; luminita.marutescu@bio.unibuc.ro (L.M.); marcela.popa@bio.unibuc.ro (M.P.)

* Correspondence: ionela-cristina.voinea@bio.unibuc.ro (I.C.V.); nicoleta.nicula@icpe-ca.ro (N.-O.N.)

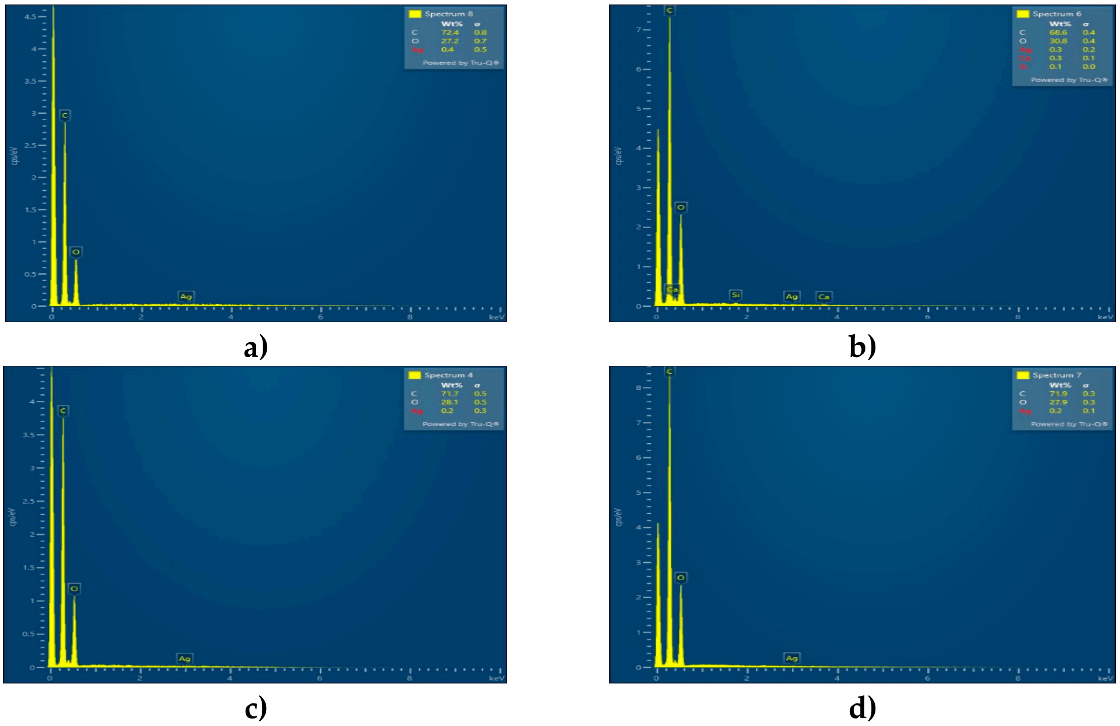

Figure S1. EDX data obtained for samples V1 (a), V2 (b), V3 (c) and V4 (d).

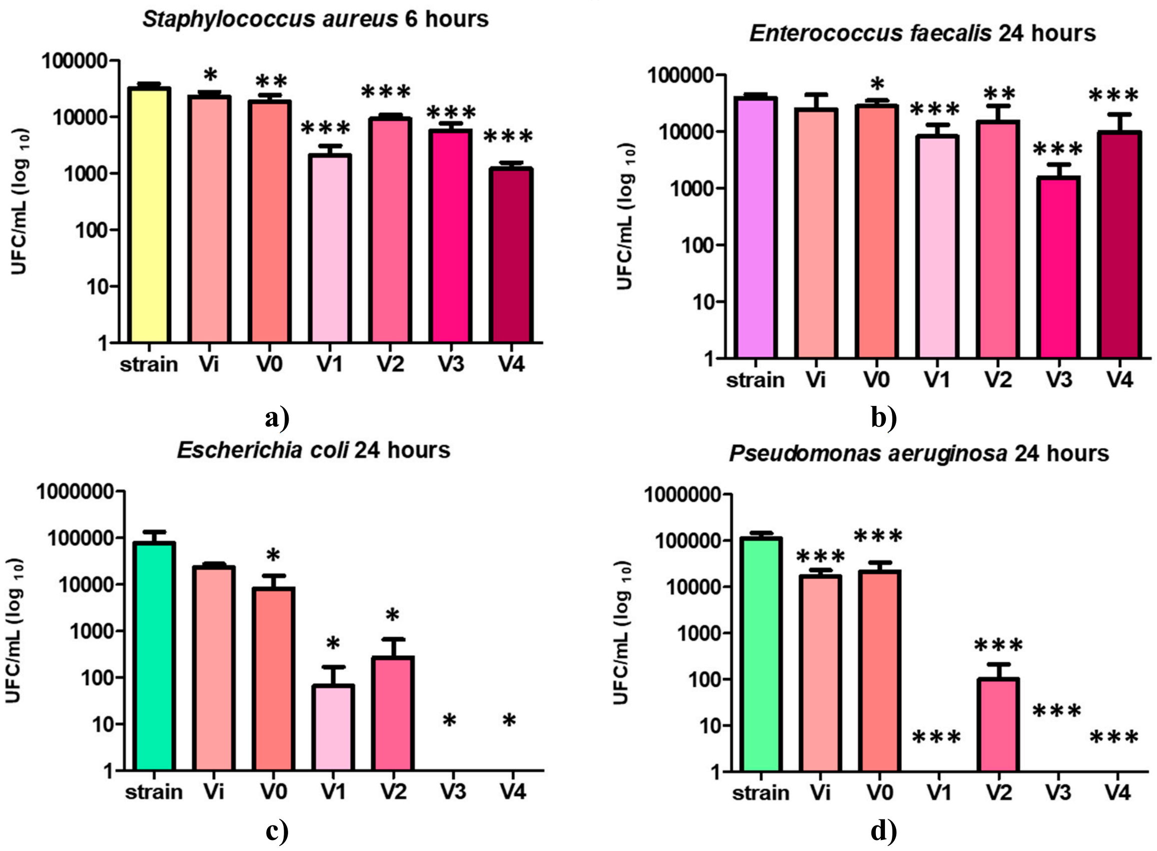

Figure S2. Viability of *Staphylococcus aureus* (a), *Enterococcus faecalis* (b), *Escherichia coli* (c) and *Pseudomonas aeruginosa* (d) in the presence of PUF/AgNPs materials. Results were calculated as the mean ± standard deviation (SD) (n = 6). * $p < 0.05$, ** $p < 0.01$ and *** $p < 0.001$ compared to strain without material.
